# Multiple Weichteilschwellungen: Computertomographie als Grundlage komplexer Tumordetektion

**DOI:** 10.1007/s00117-026-01590-x

**Published:** 2026-04-07

**Authors:** Melisa Özgüneyli, Daaje Reents, Suzan Abu Zanat, Sophia Engel

**Affiliations:** Ruhr University Bochum, Johannes Wesling Klinikum Minden, Universitätsinstitut für Radiologie, Neuroradiologie und Nuklearmedizin, Hans-Nolte-Straße 1, 32429 Minden, Deutschland

## Anamnese

Wir berichten über den Fall einer 84-jährigen Patientin, welche sich zur Abklärung einer Weichteilschwellung in stationärer Behandlung befand. Eigenanamnestisch wurden ein paroxysmales Vorhofflimmern, eine arterielle Hypertonie und eine Trigeminusneuralgie berichtet. Eine Malignomerkrankung sei nicht bekannt, und eine B‑Symptomatik bestünde nicht. Das Aufnahmelabor zeigte eine Leukozytose (14,7 G/l) und Thrombozytose (588 G/l) und war im Übrigen unauffällig.

Im Rahmen der klinischen Untersuchung konnte neben pathologisch vergrößerten Lymphknoten (LK) rechts zervikal ein auffälliger Tastbefund rechts mammär festgestellt werden, welcher weiter senologisch abgeklärt wurde. Sonographisch bestätigte sich dabei ein suspekter Herdbefund von bis zu 14 mm, welcher im Folgenden stanzbioptisch gesichert wurde. Es ergab sich die Diagnose eines Mammakarzinoms (mäßig differenziertes invasives Mammakarzinom [„no special type“, NST], Hormonrezeptor-positiv).

**Abb. 1:**
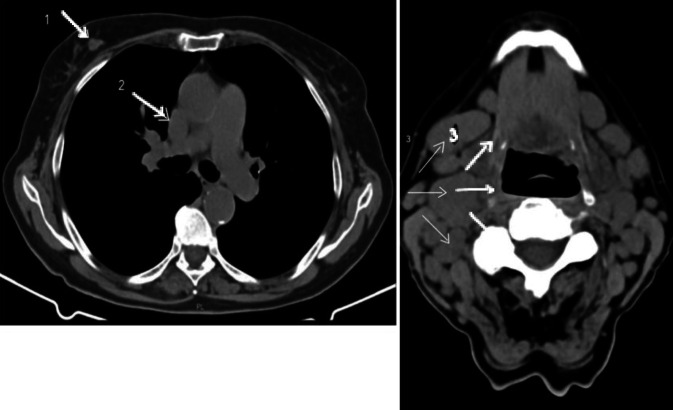
*1.* Brustherde rechts. *2.* Vergrößerte Lymphknoten (LK) mediastinal. *3.* Multiple vergrößerte LK zervikal rechts

Im Rahmen des Stagings wurde eine CT-Untersuchung von Hals, Thorax und Abdomen durchgeführt. Dabei zeigten sich neben dem bereits histologisch gesicherten Herdbefund rechts mammär korrespondierend zum klinischen Befund pathologisch vergrößerte Lymphknoten rechts zervikal sowie rechts mediastinal (Position 4R) (Abb. 1). Im interdisziplinären Konsens entschied man sich für eine histologische Sicherung dieser Befunde.

## Histologische Befunde

1. Zervikaler Lymphknoten: Diffus großzelliges B‑Zell-Lymphom. Dabei keine Hinweise auf Infiltrate durch ein Mammakarzinom.
2. Mediastinaler Lymphknoten: Follikuläres Lymphom, auch hier keine Hinweise auf Infiltrate durch das Mammakarzinom.

## Wie lautet Ihre Diagnose?

Im multidisziplinären Tumorboard wurde das weitere therapeutische Prozedere wie folgt festgelegt: Zunächst sollte die Therapie des B‑Zell-Lymphoms nach einem Mini-R-CHOP-Schema erfolgen. Parallel startete die antihormonelle Therapie des Mammakarzinoms mit einem Aromataseinhibitor.

**Diagnose:** Mammakarzinom rechts. Diffus großzelliges B‑Zell-Lymphom rechts zervikal. Follikuläres Lymphom mediastinal

Nach der Therapie des B‑Zell-Lymphoms sollte eine erneute Vorstellung im Brustzentrum zur ggf. operativen Therapie des Mammakarzinoms erfolgen.

## Definition

Das follikuläre Lymphom gehört zu den Non-Hodgkin-Lymphomen, welche aus B‑Zellen des lymphatischen Systems entstehen. Da es zu den niedrig malignen Lymphomen zählt und ein langsames Wachstum aufweist, wird in den meisten Fällen ein abwartendes Verhalten gewählt („watch and wait“) [1].

Das diffuse großzellige B‑Zell-Lymphom ist das häufigste aggressive Non-Hodgkin-Lymphom bei Erwachsenen. Es macht sich, ähnlich wie das follikuläre Lymphom, durch eine schmerzlose Lymphknotenschwellung bemerkbar. Im Gegensatz zum follikulären Lymphom zeigt es jedoch häufig ein rasches und aggressives Wachstum [2].

Etwa 30–40 % aller Patienten mit einem follikulären Lymphom entwickeln im Verlauf eine Transformation zu einem aggressiveren Lymphom, in den meisten Fällen zu einem diffusen großzelligen B‑Zell-Lymphom [3].

Das invasive Mammakarzinom NST ist die häufigste Form von Brustkrebs. Der Tumor kann Hormonrezeptor-positiv oder -negativ sein [4].

## Therapie und Verlauf

Das simultane Auftreten von Tumorerkrankungen stellt unter Berücksichtigung von Alter, Komorbiditäten, Aggressivität und Dynamik der Erkrankung sowie Neben- und Wechselwirkungen der Therapien eine besondere interdisziplinäre Herausforderung dar. Unter Abwägung von Nutzen und Risiken berücksichtigte das sequenzielle therapeutische Vorgehen die anzunehmende Aggressivität und den damit verbundenen Therapiedruck der einzelnen Erkrankungen. Als potenziell bedrohlichste Erkrankung wurde zunächst das diffuse großzellige B‑Zell-Lymphom behandelt. Aufgrund des Alters und der Komorbiditäten der Patientin wurde eine abgeschwächte Variante des R‑CHOP-Schemas (Mini-R-CHOP) eingesetzt. Bei dieser Variante, welche häufig bei älteren und gebrechlichen Patient:innen eingesetzt wird, werden die Dosierungen der Chemotherapeutika reduziert, während der therapeutische Effekt weitestgehend erhalten bleibt [2].

Eine Reevaluation einer operativen Therapie des Mammakarzinoms sollte in diesem Fall nach Abschluss der Lymphomtherapie erfolgen.

Parallel wurde unter Berücksichtigung des Alters und des Hormonrezeptorstatus der Patientin eine Therapie mit einem Aromataseinhibitor eingeleitet [4].

## Fazit

Dieser Fall ist lehrreich, da er uns in einer im klinischen Alltag nicht seltenen Konstellation eine wichtige Einschränkung der bildgebenden Diagnostik vor Augen führt. Radiologische Befunde wie Lymphknotenschwellungen oder Weichteilraumforderungen sind oftmals unspezifisch und können vielfältige Ursachen haben. Gleichzeitig ist gerade bei älteren Patient:innen das simultane Auftreten maligner Erkrankungen mit bisweilen unterschiedlicher Aggressivität keine Seltenheit. Der adäquate Umfang diagnostischer Maßnahmen und die Wahl der richtigen Therapie bzw. Therapiereihenfolge kann in diesen Fällen eine besondere interdisziplinäre Herausforderung darstellen. Vor dem Hintergrund demografischer Entwicklungen und einer wachsenden Therapielandschaft ist anzunehmen, dass es sich hierbei um eine Konstellation von zunehmender klinisch-praktischer Relevanz handelt, bei der die Radiologie eine Schlüsselrolle einnimmt.
